# 

**Published:** 1884-05

**Authors:** 


					﻿Vol. IV.
MAY, 1884.
No. 5.
IPHE
HOMEOPATHIC PHYSICIAN,
A MONTHLY JOURNAL OF MEDICAL SCIENCE.
“ Seek the Truth: come whence it may, cost what it wUl."
HL CT. LEE, IML ZD.,
EDITOR.
CONTENTS:
(See inside of Covesr.)
PHILADELPHIA:
No. 3109 CHESTNUT STREET.
USTE'W YORK:
A. L. CHATTERTON, PUBLISHING COMPANY, PUBLISHER’S AGENTS.
LON’BON:
ALFRED HEATH & CO., Sole Agents for Great Britain,
114 Ebury Street, Eaton Square.
Yearly Subscription, $2.50, invariably in advance.
Entered at the Philadelphia Post-Office as Second* Class Mail Matter.
				

